# Poly[(μ-1*H*-benzimidazole-5,6-dicarboxyl­ato)lead(II)]

**DOI:** 10.1107/S1600536811017065

**Published:** 2011-05-14

**Authors:** Jinhua Chen, Chun Zheng, Yuezhu Wang, Tingting Yun, Yifan Luo

**Affiliations:** a School of Chemistry and Environment, South China Normal University, Guangzhou 510006, People’s Republic of China

## Abstract

The crystal structure of the two-dimensional polymeric title compound, [Pb(C_9_H_4_N_2_O_4_)]_*n*_, comprises one crystallo­graphic­ally independent Pb^II^ atom and one fully deprotonated 1*H*-benzimidazole-5,6-dicarboxyl­ate (H_2_ *L*) ligand. The Pb^II^ atom is seven-coordinated by six O atoms and one N atom from the H_2_ *L* ligands, giving a capped octa­hedral coordination geometry. The structure is a layered two-dimensional coordination polymer extending parallel to (100) with N—H⋯O hydrogen bonds inter­actions between the layers, stabilizing the crystal structure.

## Related literature

For applications of metal-organic frameworks, see: Li *et al.* (2007 ▶). For related structures, see: Gao *et al.* (2008 ▶); Lo *et al.*. (2007 ▶); Wang *et al.* (2009 ▶); Wei *et al.* (2008 ▶);Yao *et al.* (2008 ▶); Zhai (2009 ▶). 
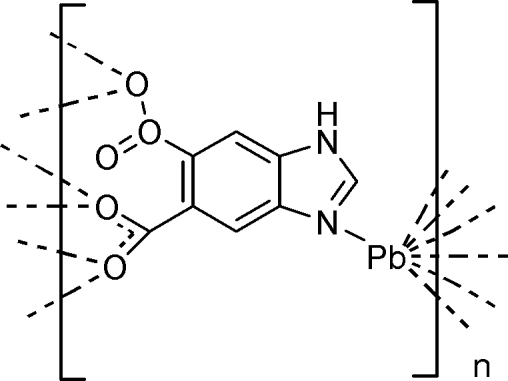


## Experimental

### 

#### Crystal data

[Pb(C_9_H_4_N_2_O_4_)]
*M* *_r_* = 411.34
Monoclinic, 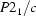

*a* = 13.127 (2) Å
*b* = 9.5571 (14) Å
*c* = 6.7557 (10) Å
β = 99.587 (2)°
*V* = 835.7 (2) Å^3^
*Z* = 4
Mo *K*α radiation
μ = 20.19 mm^−1^
*T* = 273 K
0.30 × 0.30 × 0.27 mm

#### Data collection

Bruker APEXII CCD diffractometer
Absorption correction: multi-scan (*SADABS*; Bruker, 2005 ▶) *T*_min_ = 0.003, *T*_max_ = 0.004
3954 measured reflections
1458 independent reflections
1273 reflections with *I* > 2σ(*I*)
*R*_int_ = 0.042

#### Refinement

*R*[*F* ^2^ > 2σ(*F* ^2^)] = 0.042
*wR*(*F* ^2^) = 0.120
*S* = 1.08
1458 reflections
133 parameters
12 restraints
H-atom parameters constrained
Δρ_max_ = 3.24 e Å^−3^
Δρ_min_ = −2.83 e Å^−3^

### 

Data collection: *APEX2* (Bruker, 2005 ▶); cell refinement: *SAINT* (Bruker, 2005 ▶); data reduction: *SAINT*; program(s) used to solve structure: *SHELXS97* (Sheldrick, 2008 ▶); program(s) used to refine structure: *SHELXL97* (Sheldrick, 2008 ▶); molecular graphics: *ORTEP-3 for Windows* (Farrugia, 1997 ▶); software used to prepare material for publication: *SHELXL97* and *PLATON* (Spek, 2009 ▶).

## Supplementary Material

Crystal structure: contains datablocks I, global. DOI: 10.1107/S1600536811017065/go2009sup1.cif

Structure factors: contains datablocks I. DOI: 10.1107/S1600536811017065/go2009Isup2.hkl

Additional supplementary materials:  crystallographic information; 3D view; checkCIF report

## Figures and Tables

**Table 1:** Hydrogen-bond geometry (Å, °)

*D*—H⋯*A*	*D*—H	H⋯*A*	*D*⋯*A*	*D*—H⋯*A*
N2—H2⋯O4^i^	0.86	2.02	2.723 (12)	138

Symmetry code: (i) 
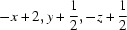
.

**Fig. 1.**
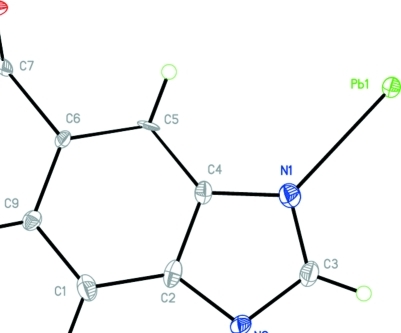
The structure of the title compound, showing the atomic numbering scheme. Non-H atoms are shown with 30% probability displacement ellipsoids.

**Fig. 2.**
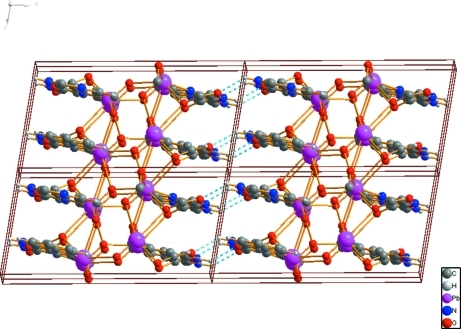
The packing diagram of the title compound, with H atoms omitted for clarity. Hydrogen bonds are shown as dashed lines.

## supplementary crystallographic information

### Comment

In recent years, metal-organic frameworks (MOF) based on supramolecular chemistry and crystal engineering have attracted extensive attention not only due to their diverse topologies and intriguing structures but also owing to their interesting physical and chemical properties, such as photoluminescence, magnetism, ferroelectricity, gas storage, ion exchange and catalysis, Li *et al.* (2007). N-Heterocyclic multicarboxylic acids have been widely used to construct MOF for their potential application. 1*H*-benzimidazole-5,6-dicarboxylic acid possesses two nitrogen atoms of imidazole ring and four oxygen atoms of carboxylate groups, and might be used as versatile linker in constructing coordination polymers with abundant hydrogen bonds. Several coordination polymers fomed by this ligand have been reported recently: Gao *et al.* (2008); Lo *et al.* (2007); Wang *et al.* (2009); Wei *et al.* (2008); Yao *et al.* (2008); Zhai (2009). Herein we report the synthesis and crystal structure of the title complex of (C_9_H_4_N_2_O_4_Pb)_n,_ Fig. 1. This is a layered 2D-coordination polymer structure with H-bonds interactions between the layers which is shown in Fig. 2.

### Experimental

A mixture of Pb(CH_3_COO)_2_ (0.6 mmol), H_2_L(0.6 mmol) and water (13 ml) was added to a 25 ml teflon-lined stainless container, which was heated to 430K and held at that temperature for 3 days. After cooling to room temperature, yellow crystals were recovered by filtration.

### Refinement

H atoms of water and hydroxyl were located in Fourier difference maps and refined with isotropic displacement parameters set at 1.5 times those of the parent O atoms. the refinement using a riding-model approximation [C–H = 0.93, O–H = 0.84 and N—H = 0.86 Å] with *U*_iso_(H) = 1.2 *U*_eq_(C,*N*) or 1.5Ueq(O).

### Crystal data

**Table:**

[Pb(C_9_H_4_N_2_O_4_)]	*F*(000) = 744.0
*M**_r_* = 411.34	*D*_x_ = 3.269 Mg m^−^^3^
Monoclinic, *P*2_1_/*c*	Mo *K*α radiation, λ = 0.71073 Å
Hall symbol: -P 2ybc	Cell parameters from 2747 reflections
*a* = 13.127 (2) Å	θ = 2.7–28.4°
*b* = 9.5571 (14) Å	µ = 20.19 mm^−^^1^
*c* = 6.7557 (10) Å	*T* = 273 K
β = 99.587 (2)°	Block, yellow
*V* = 835.7 (2) Å^3^	0.30 × 0.30 × 0.27 mm
*Z* = 4	

### Data collection

**Table:**

Bruker APEXII CCD diffractometer	1458 independent reflections
Radiation source: fine-focus sealed tube	1273 reflections with *I* > 2σ(*I*)
graphite	*R*_int_ = 0.042
φ and ω scans	θ_max_ = 25.0°, θ_min_ = 2.7°
Absorption correction: multi-scan (*SADABS*; Bruker, 2005)	*h* = −15→15
*T*_min_ = 0.003, *T*_max_ = 0.004	*k* = −11→8
3954 measured reflections	*l* = −7→8

### Refinement

**Table:**

Refinement on *F*^2^	Primary atom site location: structure-invariant direct methods
Least-squares matrix: full	Secondary atom site location: difference Fourier map
*R*[*F*^2^ > 2σ(*F*^2^)] = 0.042	Hydrogen site location: inferred from neighbouring sites
*wR*(*F*^2^) = 0.120	H-atom parameters constrained
*S* = 1.08	*w* = 1/[σ^2^(*F*_o_^2^) + (0.0814*P*)^2^] where *P* = (*F*_o_^2^ + 2*F*_c_^2^)/3
1458 reflections	(Δ/σ)_max_ = 0.001
133 parameters	Δρ_max_ = 3.24 e Å^−^^3^
12 restraints	Δρ_min_ = −2.83 e Å^−^^3^

### Special details

**Table:**

Geometry. All e.s.d.'s (except the e.s.d. in the dihedral angle between two l.s. planes) are estimated using the full covariance matrix. The cell e.s.d.'s are taken into account individually in the estimation of e.s.d.'s in distances, angles and torsion angles; correlations between e.s.d.'s in cell parameters are only used when they are defined by crystal symmetry. An approximate (isotropic) treatment of cell e.s.d.'s is used for estimating e.s.d.'s involving l.s. planes.
Refinement. Refinement of *F*^2^ against ALL reflections. The weighted *R*-factor *wR* and goodness of fit *S* are based on *F*^2^, conventional *R*-factors *R* are based on *F*, with *F* set to zero for negative *F*^2^. The threshold expression of *F*^2^ > σ(*F*^2^) is used only for calculating *R*-factors(gt) *etc*. and is not relevant to the choice of reflections for refinement. *R*-factors based on *F*^2^ are statistically about twice as large as those based on *F*, and *R*- factors based on ALL data will be even larger. The number of independent reflections and the number of reflections used in the refinement are not the same, because we use 'omit -3 50' to enhance the'_diffrn_measured_fraction_theta_full'.

### Fractional atomic coordinates and isotropic or equivalent isotropic displacement parameters (Å^2^)

**Table:**

	*x*	*y*	*z*	*U*_iso_*/*U*_eq_	
C4	0.7560 (4)	0.3072 (4)	0.2539 (9)	0.019 (2)	
C5	0.6729 (3)	0.2152 (5)	0.2241 (10)	0.0164 (19)	
H5	0.6058	0.2496	0.1959	0.020*	
C6	0.6901 (4)	0.0717 (4)	0.2362 (9)	0.0152 (18)	
C9	0.7904 (4)	0.0203 (4)	0.2783 (9)	0.0169 (19)	
C1	0.8736 (3)	0.1123 (6)	0.3081 (9)	0.025 (3)	
H1	0.9407	0.0779	0.3363	0.030*	
C2	0.8564 (4)	0.2558 (5)	0.2959 (9)	0.021 (2)	
Pb1	0.62381 (3)	0.62780 (3)	0.17812 (5)	0.0172 (2)	
N1	0.7597 (7)	0.4523 (8)	0.2560 (13)	0.0252 (19)	
C3	0.8566 (8)	0.4844 (11)	0.2927 (15)	0.025 (2)	
H3	0.8810	0.5759	0.3003	0.029*	
N2	0.9199 (8)	0.3693 (7)	0.3195 (14)	0.021 (2)	
H2	0.9863	0.3690	0.3459	0.025*	
C8	0.8123 (9)	−0.1314 (8)	0.3226 (17)	0.020 (2)	
O4	0.8944 (6)	−0.1832 (8)	0.2949 (13)	0.036 (2)	
C7	0.6000 (7)	−0.0282 (8)	0.1935 (12)	0.0146 (19)	
O2	0.5921 (6)	−0.1031 (6)	0.0387 (11)	0.0220 (16)	
O1	0.5389 (5)	−0.0325 (6)	0.3196 (9)	0.0184 (14)	
O3	0.7406 (4)	−0.2011 (6)	0.3876 (9)	0.0167 (13)	

### Atomic displacement parameters (Å^2^)

**Table:**

	*U*^11^	*U*^22^	*U*^33^	*U*^12^	*U*^13^	*U*^23^
C4	0.024 (5)	0.013 (4)	0.019 (5)	−0.001 (4)	0.002 (4)	0.001 (3)
C5	0.006 (4)	0.021 (4)	0.022 (4)	0.005 (3)	0.002 (3)	0.002 (3)
C6	0.014 (5)	0.012 (4)	0.021 (5)	−0.003 (3)	0.007 (4)	0.000 (3)
C9	0.018 (5)	0.017 (4)	0.017 (5)	−0.003 (4)	0.007 (4)	−0.001 (3)
C1	0.030 (6)	0.017 (5)	0.030 (6)	0.005 (3)	0.011 (5)	0.001 (3)
C2	0.033 (6)	0.015 (5)	0.016 (5)	−0.006 (4)	0.008 (4)	−0.002 (4)
Pb1	0.0188 (3)	0.0151 (3)	0.0172 (3)	−0.00085 (11)	0.0019 (2)	−0.00061 (10)
N1	0.026 (5)	0.021 (4)	0.029 (5)	0.001 (4)	0.008 (4)	−0.003 (3)
C3	0.029 (6)	0.016 (5)	0.028 (6)	−0.004 (4)	0.003 (5)	−0.001 (4)
N2	0.016 (5)	0.019 (4)	0.031 (5)	−0.004 (3)	0.010 (4)	−0.001 (3)
C8	0.013 (6)	0.019 (5)	0.027 (6)	0.001 (3)	0.003 (5)	−0.006 (3)
O4	0.021 (4)	0.021 (4)	0.066 (6)	0.000 (3)	0.011 (4)	0.004 (4)
C7	0.013 (5)	0.014 (4)	0.014 (5)	0.004 (3)	−0.006 (4)	0.005 (3)
O2	0.030 (4)	0.017 (3)	0.021 (4)	0.003 (3)	0.010 (3)	−0.003 (3)
O1	0.010 (3)	0.032 (3)	0.013 (3)	−0.001 (2)	0.003 (3)	0.002 (2)
O3	0.010 (3)	0.018 (3)	0.021 (3)	0.001 (2)	0.000 (3)	0.003 (2)

### Geometric parameters (Å, °)

**Table:**

C4—N1	1.387 (8)	Pb1—O1^iv^	2.653 (6)
C4—C5	1.389 (7)	Pb1—O2^i^	2.746 (6)
C4—C2	1.391 (7)	N1—C3	1.292 (13)
C5—C6	1.390 (7)	C3—N2	1.371 (13)
C5—H5	0.9300	C3—H3	0.9300
C6—C9	1.390 (7)	N2—H2	0.8600
C6—C7	1.510 (10)	C8—O4	1.228 (13)
C9—C1	1.390 (7)	C8—O3	1.289 (11)
C9—C8	1.498 (9)	C7—O2	1.258 (11)
C1—C2	1.390 (7)	C7—O1	1.265 (11)
C1—H1	0.9300	O2—Pb1^iv^	2.549 (7)
C2—N2	1.362 (9)	O2—Pb1^v^	2.746 (6)
Pb1—N1	2.442 (8)	O1—Pb1^vi^	2.630 (6)
Pb1—O3^i^	2.511 (6)	O1—Pb1^ii^	2.653 (6)
Pb1—O2^ii^	2.549 (7)	O3—Pb1^v^	2.511 (6)
Pb1—O1^iii^	2.630 (6)		
			
N1—C4—C5	131.3 (5)	N1—Pb1—O2^i^	141.3 (2)
N1—C4—C2	108.7 (5)	O3^i^—Pb1—O2^i^	68.1 (2)
C5—C4—C2	120.0 (4)	O2^ii^—Pb1—O2^i^	112.01 (18)
C4—C5—C6	120.0 (4)	O1^iii^—Pb1—O2^i^	118.1 (2)
C6—C5—H5	120.0	O1^iv^—Pb1—O2^i^	89.52 (19)
C4—C5—H5	120.0	C3—N1—C4	105.7 (7)
C9—C6—C5	120.0 (4)	C3—N1—Pb1	122.6 (6)
C9—C6—C7	120.0 (4)	C4—N1—Pb1	131.4 (5)
C5—C6—C7	119.9 (4)	N1—C3—N2	113.0 (9)
C1—C9—C6	120.1 (4)	N1—C3—H3	123.5
C1—C9—C8	117.6 (5)	N2—C3—H3	123.5
C6—C9—C8	121.8 (5)	C2—N2—C3	106.1 (8)
C2—C1—H1	120.0	C2—N2—H2	126.9
C9—C1—H1	120.0	C3—N2—H2	126.9
C9—C1—C2	120.0 (4)	O4—C8—O3	123.6 (8)
N2—C2—C1	133.6 (5)	O4—C8—C9	120.2 (8)
N2—C2—C4	106.4 (5)	O3—C8—C9	116.2 (8)
C1—C2—C4	120.0 (4)	O2—C7—O1	124.6 (8)
N1—Pb1—O3^i^	88.3 (2)	O2—C7—C6	118.2 (8)
N1—Pb1—O2^ii^	87.7 (2)	O1—C7—C6	117.2 (7)
O3^i^—Pb1—O2^ii^	72.7 (2)	C7—O2—Pb1^iv^	147.5 (6)
N1—Pb1—O1^iii^	99.4 (2)	C7—O2—Pb1^v^	105.1 (5)
O3^i^—Pb1—O1^iii^	142.76 (18)	Pb1^iv^—O2—Pb1^v^	101.6 (2)
O2^ii^—Pb1—O1^iii^	71.3 (2)	C7—O1—Pb1^vi^	126.3 (5)
N1—Pb1—O1^iv^	98.2 (2)	C7—O1—Pb1^ii^	114.1 (5)
O3^i^—Pb1—O1^iv^	149.59 (19)	Pb1^vi^—O1—Pb1^ii^	114.4 (2)
O2^ii^—Pb1—O1^iv^	136.9 (2)	C8—O3—Pb1^v^	123.8 (6)
O1^iii^—Pb1—O1^iv^	65.6 (2)		
			
N1—C4—C5—C6	−177.8 (6)	O1^iii^—Pb1—N1—C4	14.3 (7)
C2—C4—C5—C6	0.0	O1^iv^—Pb1—N1—C4	−52.2 (7)
C4—C5—C6—C9	0.0	O2^i^—Pb1—N1—C4	−151.8 (5)
C4—C5—C6—C7	−176.9 (6)	C4—N1—C3—N2	−1.0 (11)
C5—C6—C9—C1	0.0	Pb1—N1—C3—N2	−175.9 (7)
C7—C6—C9—C1	176.9 (6)	C1—C2—N2—C3	−178.7 (6)
C5—C6—C9—C8	170.9 (7)	C4—C2—N2—C3	0.4 (9)
C7—C6—C9—C8	−12.2 (8)	N1—C3—N2—C2	0.4 (12)
C6—C9—C1—C2	0.0	C1—C9—C8—O4	−32.8 (12)
C8—C9—C1—C2	−171.2 (7)	C6—C9—C8—O4	156.1 (8)
C9—C1—C2—N2	179.1 (9)	C1—C9—C8—O3	147.9 (7)
C9—C1—C2—C4	0.0	C6—C9—C8—O3	−23.2 (11)
N1—C4—C2—N2	−1.1 (7)	C5—C6—C7—O2	112.1 (7)
C5—C4—C2—N2	−179.3 (6)	C9—C6—C7—O2	−64.9 (8)
N1—C4—C2—C1	178.2 (5)	C5—C6—C7—O1	−69.5 (8)
C5—C4—C2—C1	0.0	C9—C6—C7—O1	113.6 (7)
C5—C4—N1—C3	179.3 (6)	O1—C7—O2—Pb1^iv^	150.4 (8)
C2—C4—N1—C3	1.3 (8)	C6—C7—O2—Pb1^iv^	−31.3 (15)
C5—C4—N1—Pb1	−6.5 (9)	O1—C7—O2—Pb1^v^	−65.2 (9)
C2—C4—N1—Pb1	175.5 (5)	C6—C7—O2—Pb1^v^	113.1 (6)
O3^i^—Pb1—N1—C3	−29.0 (8)	O2—C7—O1—Pb1^vi^	−94.0 (9)
O2^ii^—Pb1—N1—C3	−101.8 (8)	C6—C7—O1—Pb1^vi^	87.7 (7)
O1^iii^—Pb1—N1—C3	−172.4 (7)	O2—C7—O1—Pb1^ii^	113.2 (8)
O1^iv^—Pb1—N1—C3	121.2 (8)	C6—C7—O1—Pb1^ii^	−65.2 (7)
O2^i^—Pb1—N1—C3	21.6 (10)	O4—C8—O3—Pb1^v^	−74.3 (13)
O3^i^—Pb1—N1—C4	157.6 (7)	C9—C8—O3—Pb1^v^	105.0 (7)
O2^ii^—Pb1—N1—C4	84.8 (7)		

Symmetry codes: (i) *x*, *y*+1, *z*; (ii) *x*, −*y*+1/2, *z*+1/2; (iii) −*x*+1, *y*+1/2, −*z*+1/2; (iv) *x*, −*y*+1/2, *z*−1/2; (v) *x*, *y*−1, *z*; (vi) −*x*+1, *y*−1/2, −*z*+1/2.

### Hydrogen-bond geometry (Å, °)

**Table:**

*D*—H···*A*	*D*—H	H···*A*	*D*···*A*	*D*—H···*A*
N2—H2···O4^vii^	0.86	2.02	2.723 (12)	138

Symmetry codes: (vii) −*x*+2, *y*+1/2, −*z*+1/2.
